# Supplementary material for: Global Proteome Profiling Revealed the Adaptive Reprogramming of Barley Flag Leaf to Drought and Elevated Temperature
Source: Cells. 2023 Jun 22;12(13):1685. doi: 10.3390/cells12131685 (PMC10340373; doi:10.3390/cells12131685)
Supplement: Supplementary file 1 [file cells-12-01685-s001.zip › Figure S1.pdf]

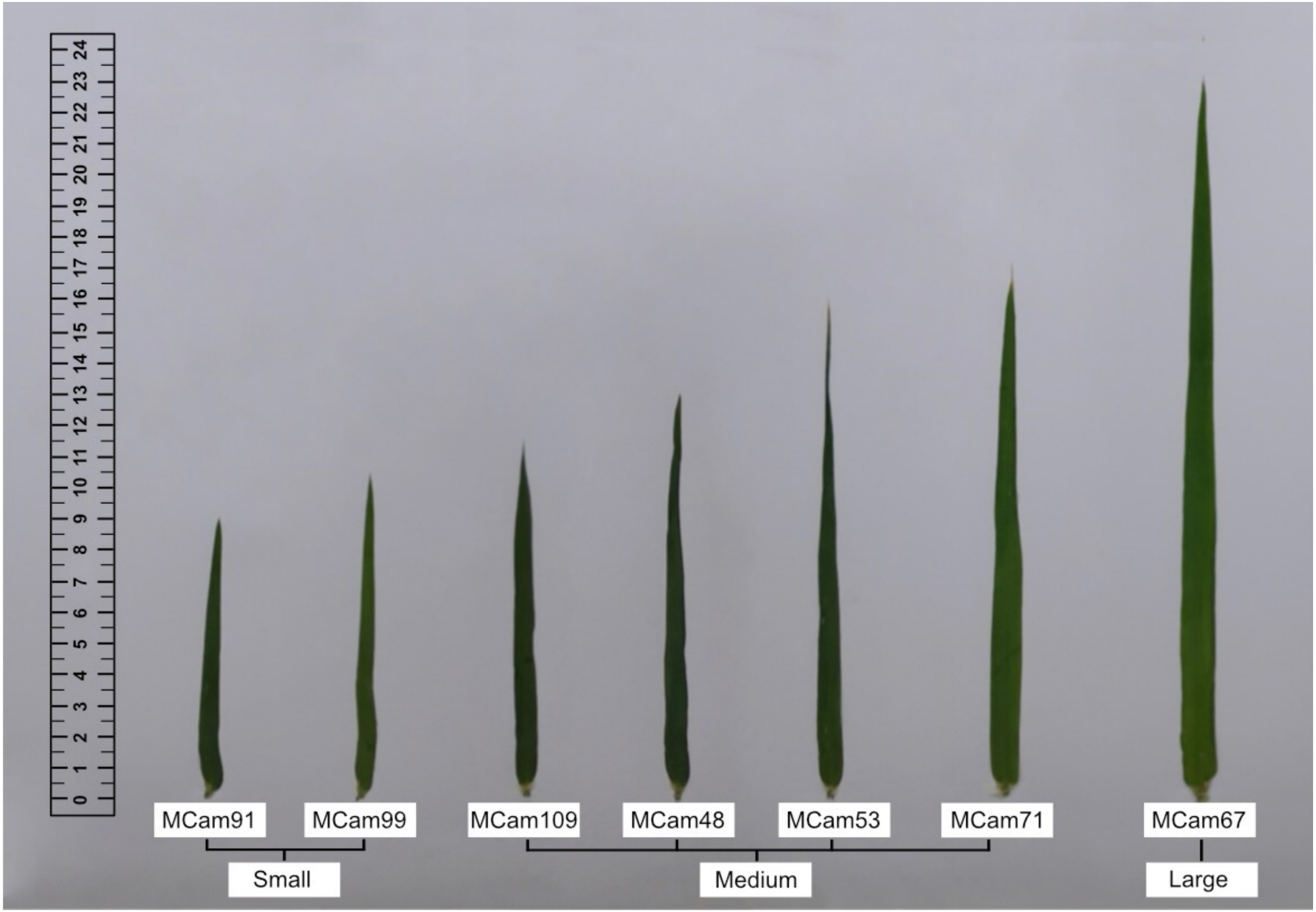

Figure S1. Classification of seven barley accession into three groups according to flag leaf size: Small, Medium, and Large (Mikołajczak et al. [22])
