# Supplementary material for: Global Proteome Profiling Revealed the Adaptive Reprogramming of Barley Flag Leaf to Drought and Elevated Temperature
Source: Cells. 2023 Jun 22;12(13):1685. doi: 10.3390/cells12131685 (PMC10340373; doi:10.3390/cells12131685)
Supplement: Supplementary file 1 [file cells-12-01685-s001.zip › Figure S2.pdf]

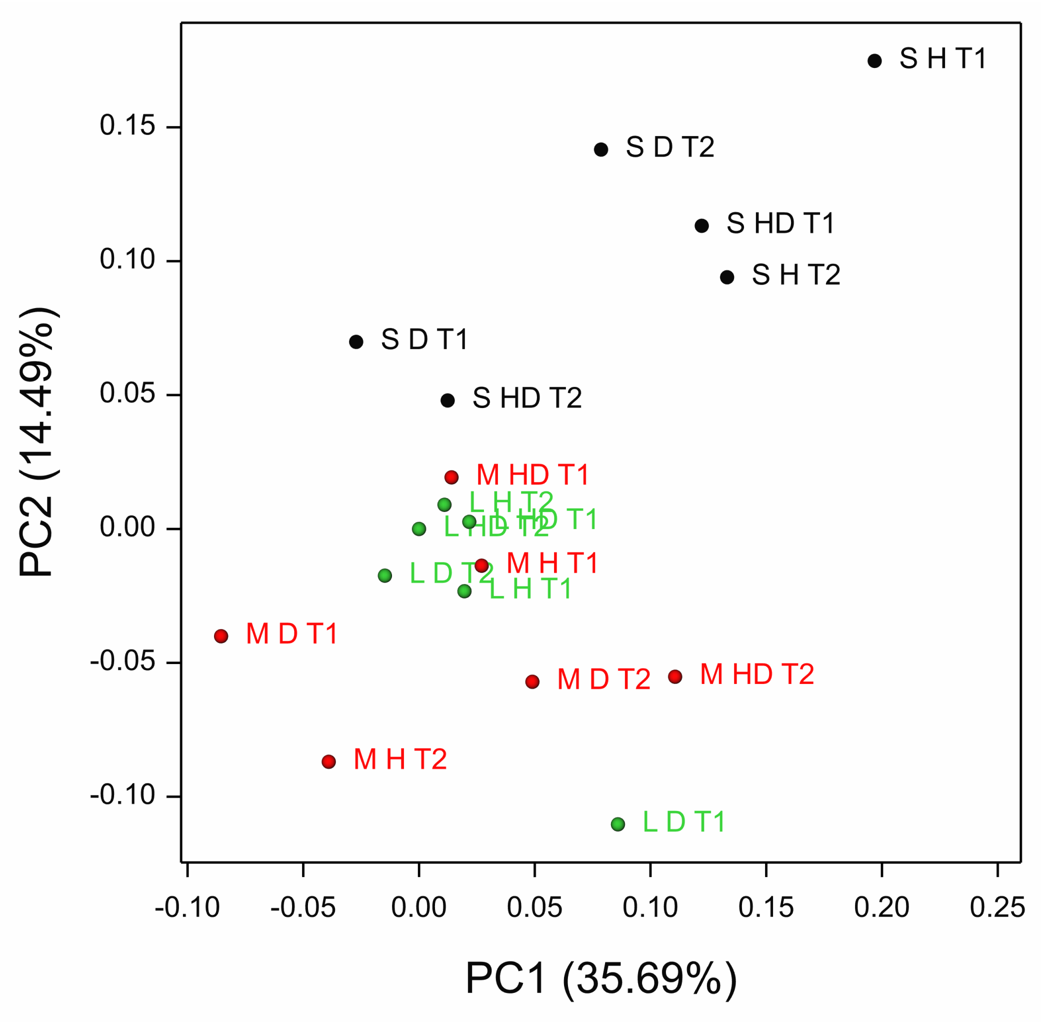

Figure S2. Principal coordinate analysis of log_2(FC) values for differentially expressed proteins with a significant change in at least two comparisons. Points represent profiles of reaction of DEPs under conditions D, H, or HD relative to control, in genotypes with small (S, black), medium (M, red) or large (L, green) flag leaves, at time point T1 or T2
